# Supplementary material for: Human pre-valvular endocardial cells derived from pluripotent stem cells recapitulate cardiac pathophysiological valvulogenesis
Source: Nat Commun. 2019 Apr 26;10:1929. doi: 10.1038/s41467-019-09459-5 (PMC6486645; doi:10.1038/s41467-019-09459-5)
Supplement: Supplementary file 1 — Supplementary Information [file 41467_2019_9459_MOESM1_ESM.pdf]

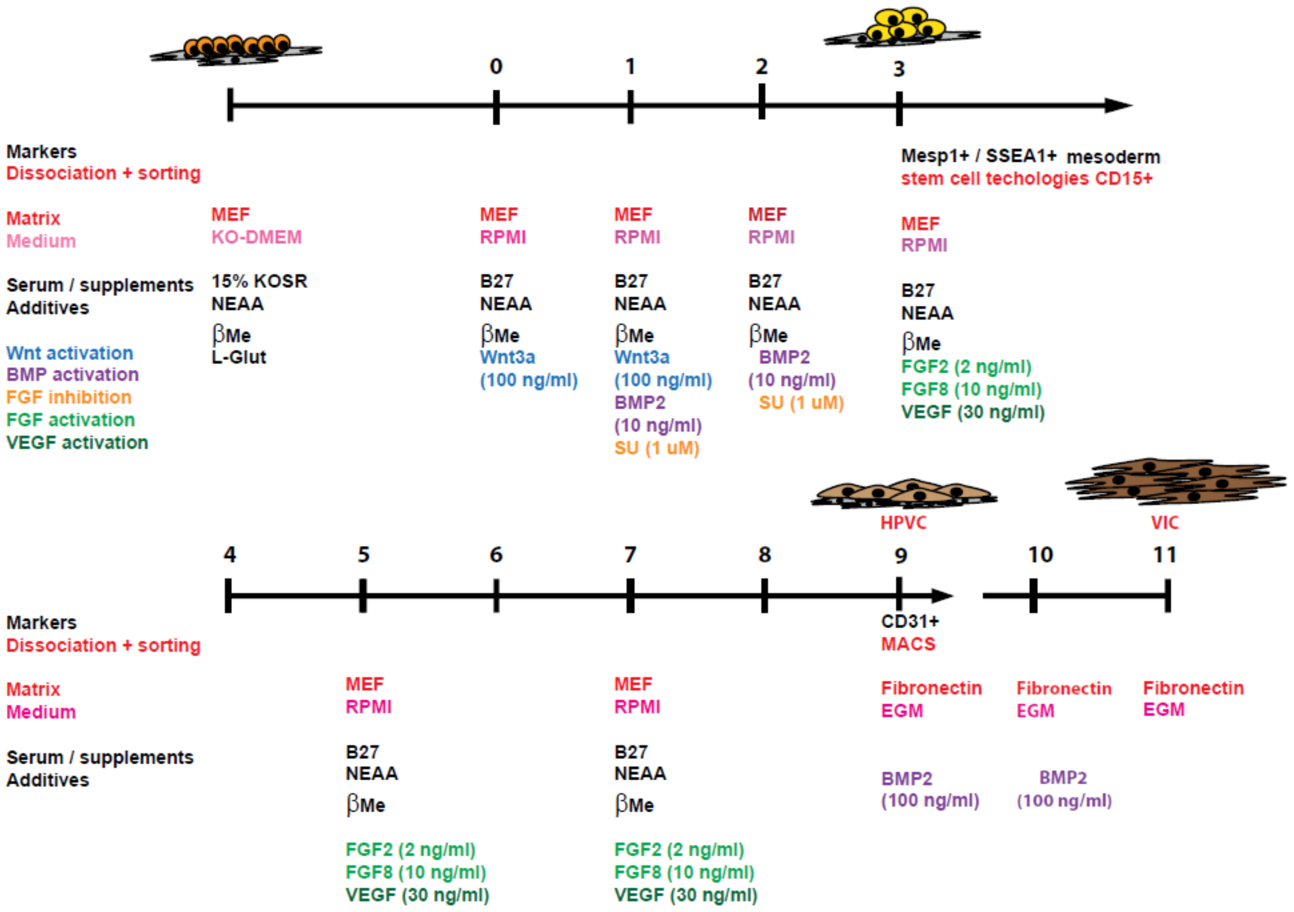

Supplementary Figure 1: schema of valvular cell differentiation
(βMe: b-mercaptoethanol; NEAA: Non Essential Amino Acid; ECGM endothelial cell growth medium)

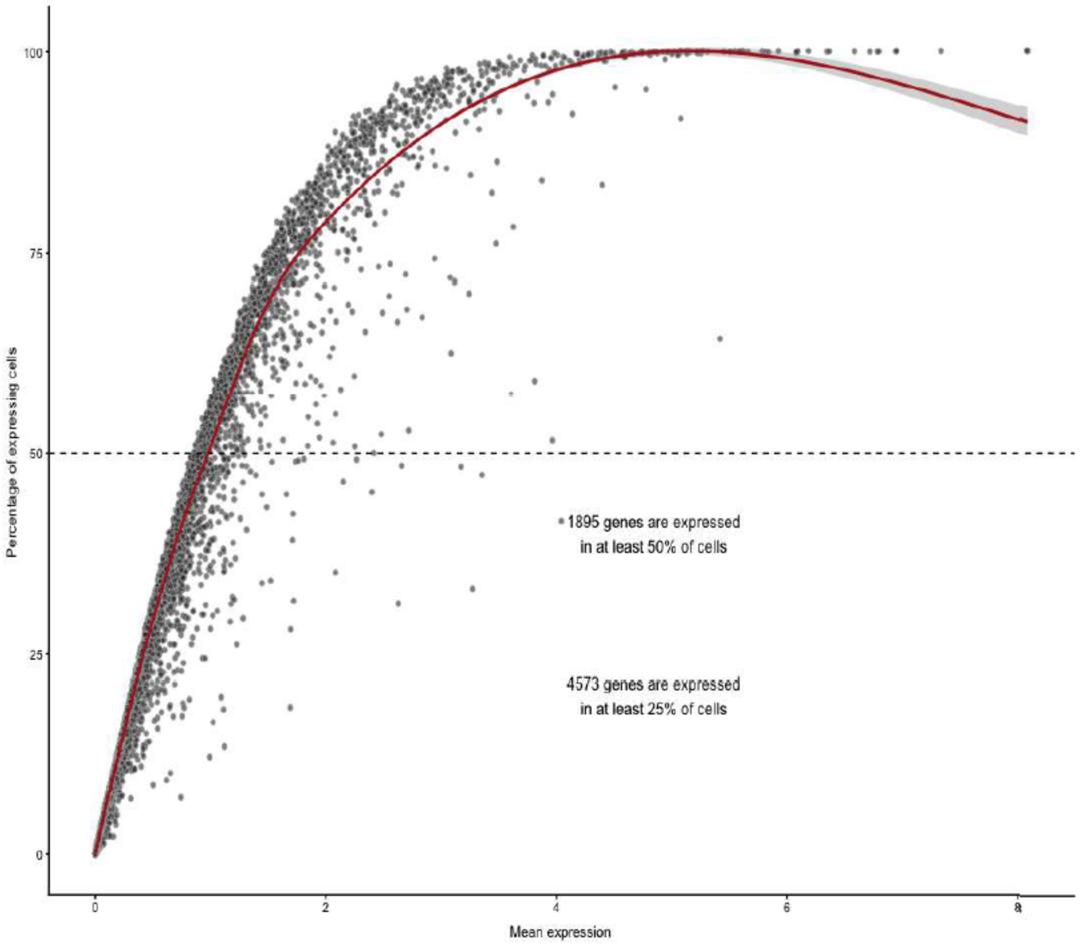

Supplementary Figure 2: number and mean expression of genes in single cell datasets

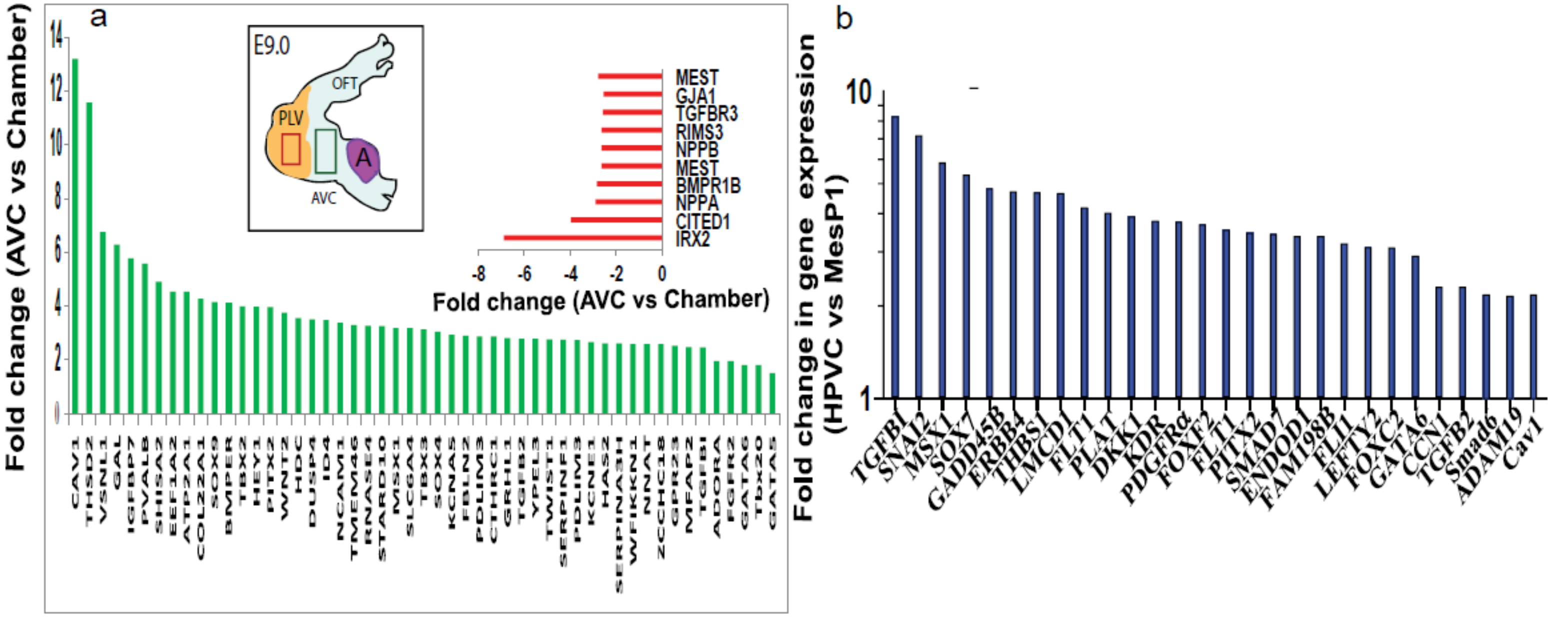

Supplementary Figure 3: Gene expression profiles of embryonic AVC endocardium and human valve progenitors.
(a)Mouse embryonic E9.0 AVCs and ventricles were dissected, RNA extracted and cRNAs used for microarrays.
The bargraph shows the most enriched genes in AVC endocardium (green bars) and the less enriched (red bars in inset) versus the chambers.
(b)SSEA-1+-sorted human MESP1+ cells were seeded on MEFs and treated with 10 ng/ml FGF8 and 30 ng/ml VEGF for 6 days.
RNA was then collected and cDNAs of FGF8/VEGF treated cells (n=3 experiments) were used for microarrays and normalized versus MESP1+ cells from the same respective cell sorting (mean±SEM). Data are normalized to 1 as the level of gene expression in SSEA1+MESP1+ cells

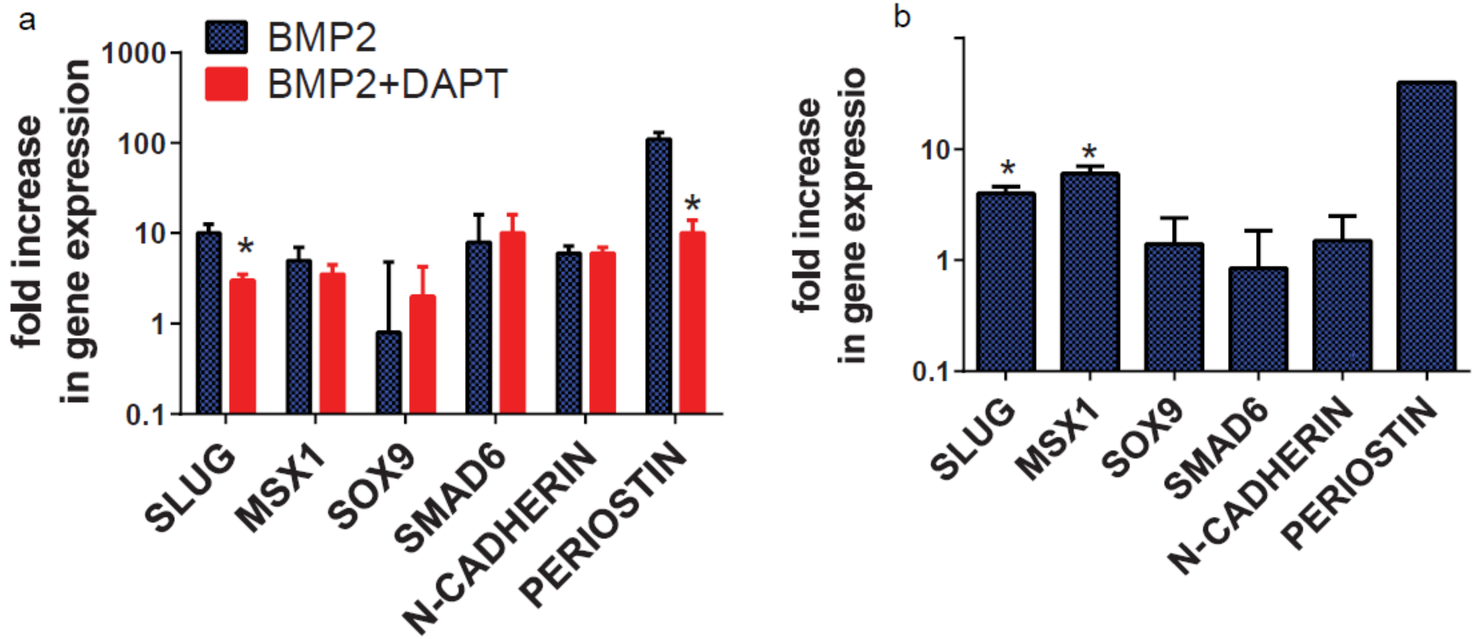

Supplementary figure 4: EMT of HPVC cells is regulated by Notch.

(a) After 1 week of FGF8/VEGF treatment on MEFs, valve progenitors (HPVCs) were recovered with trypsin, seeded on fibronectin coated wells and treated with 100 ng/ml BMP2 in the presence or absence of the notch inhibitor DAPT, while control cells were seeded on fibronectin and treated with VEGF to stop EMT. After 3-5 days, RNA was recovered and cDNAs were run in real-time PCR for post EMT markers. BMP2 samples are normalized on control (before treatment) samples, showing an increase in the expression of post-EMT markers (*) significantly different $p \leq 0.01$, n=5.

(b) Control FGF8/VEGF treated cells were transfected with NCDI, the notch intracellular domain, or empty backbone vector (mock), and gene expression was monitored three days later by real-time PCR and expressed as fold increase over the mock.(*) significantly different $p \leq 0.01$, n=7 experiments

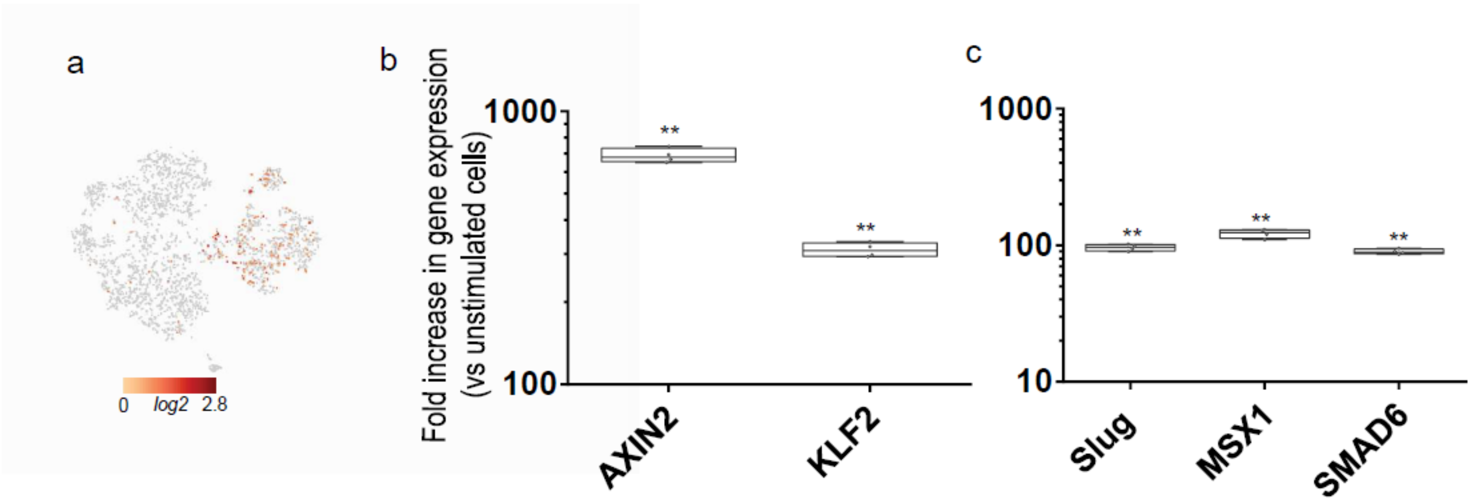

Supplementary figure 5: Wnt activation of Klf2
(a) single cell-sequencing of HPVCs: cluster of Klf2+ cells (b) wnt induced KLF2 and (c) EMT genes in HPVCs.
Confluent CD31+-sorted HPVC were challenged for 24H with wnt3a and spondin3 and RNA extracted for RT-Q-PCR
(2 experiments performed in duplicate, ** significantly different $p \leq 0.01$)

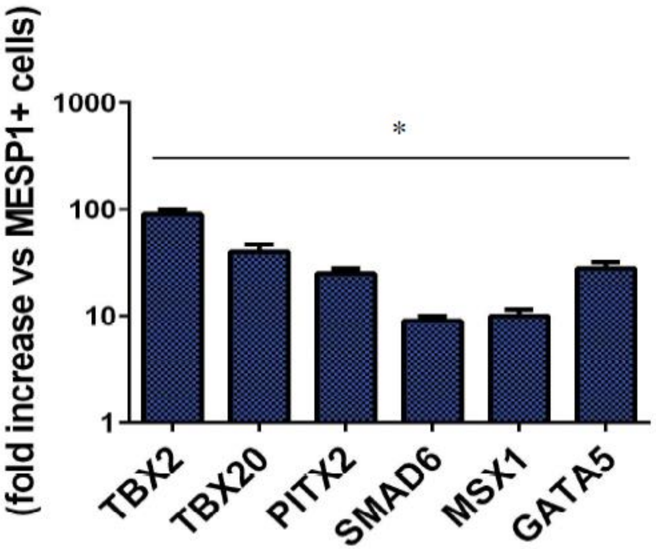

Supplementary figure 6: Gene expression of iPS cells-derived HPVCs.
Data are expressed as fold increase versus SSEA1+/MESP1+ sorted cells
(n=5, mean ± SEM; * statistically significant p≤ 0.01 from expression in SSEA1+/MESP1+ cells)

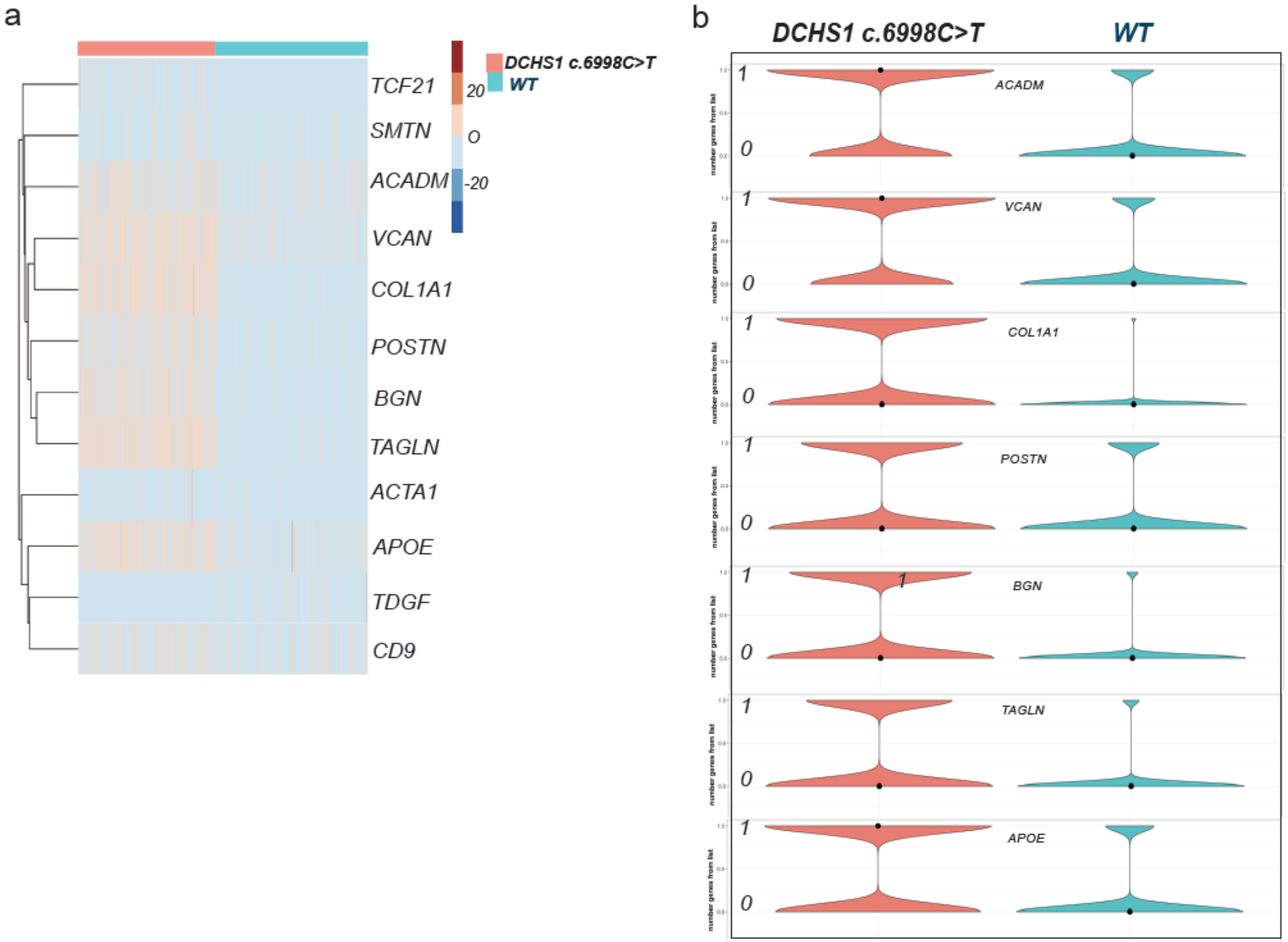

Supplementary figure 7: Differential expression of genes between wt and DCSHS 1 mutated cells
comparative heat map (a) and violin plots (b) between wt and DCSHS 1 mutated cells

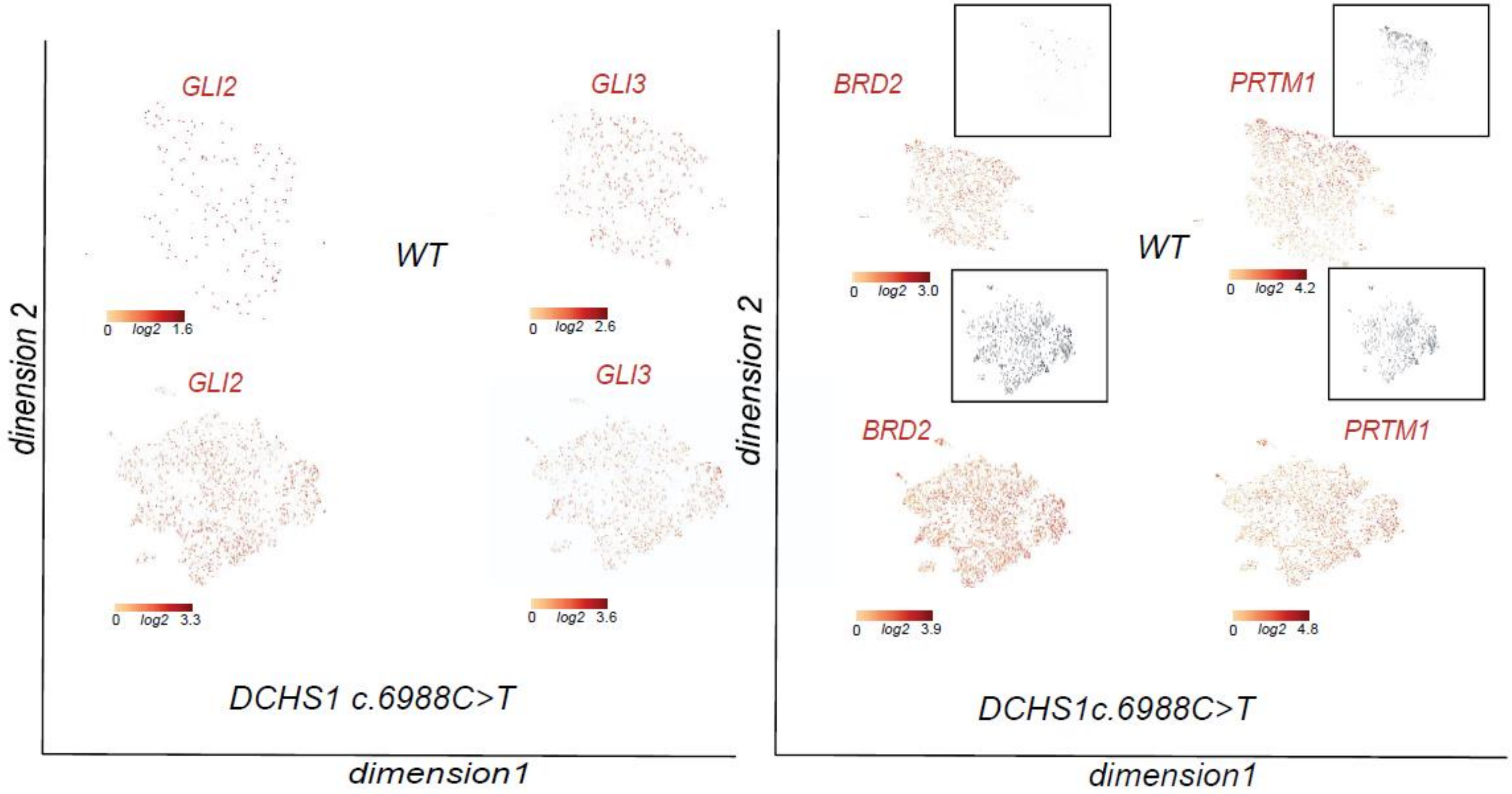

Supplementary figure 8 : SHH pathway

t-distributed stochastic neighbor embedding (t-SNE) 2D cell map 10X genomics (n=4316 cells) Highlight of cell populations expressing GLI2 and GLI3 (left panel) and BRD2 and PRTM1 (right panel) in both wt and DCHS1 c.6988C >T valvular cells . Insets show cells with high expression of BRD2 and PRTM1 (fold change log2>2).
