# Supplementary material for: Human pre-valvular endocardial cells derived from pluripotent stem cells recapitulate cardiac pathophysiological valvulogenesis
Source: Nat Commun. 2019 Apr 26;10:1929. doi: 10.1038/s41467-019-09459-5 (PMC6486645; doi:10.1038/s41467-019-09459-5)
Supplement: Supplementary file 4 — Description of Additional Supplementary Files [file 41467_2019_9459_MOESM4_ESM.docx]

**Title:** Supplementary data 1
**Description:** Single cell RNA sequencing: Data of wt HPVCs, wt post-EMT VICs , DCHS1 c.6988C >T postEMT VICs (all clusters, all genes)

**Title:** Supplementary data 2
**Description:** Primer sequences.
